# A systematic review of published antimalarial clinical trials: parasite clearance of artemisinin-containing regimens in the treatment of uncomplicated malaria

**DOI:** 10.1186/1475-2875-11-S1-P37

**Published:** 2012-10-15

**Authors:** Debashish Das, Delia Bethell, Richard Cooksey, Finn Anderson, Patranuch Sapchookul, Patrice Piola, Philippe J Guerin, Ric N Price, Kasia Stepniewska

**Affiliations:** 1 Worldwide Antimalarial Resistance Network (WWARN), Asia Regional Centre, Bangkok, Thailand; 2 WorldWide Antimalarial Resistance Network (WWARN), University of Oxford, UK; 3 Global Health Division, Menzies School of Health Research and Charles Darwin University, Darwin, Northern Territory, Australia; 4 Centre for Tropical Medicine, Nuffield Department of Clinical Medicine, University of Oxford, UK

## Background

Parasitaemia on day 3 has been proposed as useful alert of potential artemisinin resistance, however, the normal variation of parasite clearance observed in ACT clinical trials is poorly documented. We reviewed the trends in parasite clearance following treatment with an artemisinin antimalarial regimen.

## Methods

A PubMed literature search identified all studies of uncomplicated falciparum malaria published between January 2000 and December 2012. References were individually reviewed to identify clinical efficacy studies. Data from clinical studies using an artemisinin derivative were extracted and entered into a Microsoft Office Access database for analysis.

## Results

In total 65,078 patients were enrolled into 213 clinical trials of artemisinin-containing regimens with 413 treatment arms containing either an artemisinin derivative alone (n=26) or in combination with a partner drug (n=387). The proportion of patients remaining parasitaemic at 24, 48 and 72 hours was documented in 115 (28%), 167 (40%) and 153 (37%) treatment arms, respectively. Excluding resistance studies in Cambodia, the median proportion of patients still parasitaemic was 53.8% [range 3-95, IQR=30.5-69.2] at 24 hours, 6% [range 0-65.9, IQR=2-11.5] at 48 hours and 0 [range 0-12.6, IQR=0-2] at 72 hours. Comparing studies from 2000-2005 and 2006-2011, the median proportion of patients remaining parasitaemic at 72 hours decreased in Africa (1.6% vs. 0, p=0.0004), but increased in Asia (0.8% vs. 1.2%, p=<0.0001). Overall in 95% of these studies the proportion of patients with peripheral parasitaemia was <6% at 72 hours.

## Conclusion

These results highlight a normal range of parasite clearance times that will underpin a surveillance system based on day 3 positivity and the impact of heterogeneity in study design, host and parasite factors. Greater understanding of factors influencing parasite clearance will come from analysis of pooled data from individual patient records.

